# Shear wave speed measurement bias in a viscoelastic phantom across six ultrasound elastography systems: a comparative study with transient elastography and magnetic resonance elastography

**DOI:** 10.1007/s10396-022-01190-x

**Published:** 2022-01-21

**Authors:** Riwa Kishimoto, Mikio Suga, Masashi Usumura, Hiroko Iijima, Masahiro Yoshida, Hiroyuki Hachiya, Tsuyoshi Shiina, Makoto Yamakawa, Kei Konno, Takayuki Obata, Tadashi Yamaguchi

**Affiliations:** 1 Applied MRI Research, Department of Molecular Imaging and Theranostics, National Institutes for Quantum Science and Technology, Chiba, Japan; 2 Center for Frontier Medical Engineering, Chiba University, 1-33 Yayoi, Inage, Chiba, Chiba 263-8522 Japan; 3 Graduate School of Science and Engineering, Chiba University, Chiba, Japan; 4 Ultrasound Imaging Center, Hyogo College of Medicine, Nishinomiya, Hyogo Japan; 5 School of Engineering, Tokyo Institute of Technology, Meguro, Tokyo Japan; 6 Graduate School of Medicine, Kyoto University, Kyoto, Japan; 7 Clinical Laboratory Medicine, Jichi Medical University School of Medicine, Shimotsuke, Tochigi Japan

**Keywords:** Ultrasound elastography, Magnetic resonance elastography, Phantom, Quantitative imaging biomarker alliance, Viscoelasticity

## Abstract

**Purpose:**

To quantify the bias of shear wave speed (SWS) measurements in a viscoelastic phantom across six different ultrasound (US) systems and to compare the SWS with those from transient elastography (TE) and magnetic resonance elastography (MRE).

**Methods:**

A viscoelastic phantom of stiffness representing fibrotic liver or healthy thyroid was measured with nine (linear probe) and 10 (convex probe) modes of six different US-based shear wave elastography (SWE) systems using linear and convex probes. SWS measurements of three regions of interest were repeated thrice at two focal depths, coupling the probe to the phantom using a jig. An MRE system using three motion-encoding gradient frequencies of 60, 90, and 120 Hz and TE were also used to measure the stiffness of the phantom.

**Results:**

The SWS from different SWE systems had mean coefficients of variation of 9.0–9.2% and 5.4–5.6% with linear and convex probes, respectively, in viscoelastic phantom measurement. The focal depth was a less significant source of SWS variability than the system. The total average SWS obtained with US-SWE systems was 19.9% higher than that obtained with MRE at 60 Hz, which is commonly used in clinical practice, and 31.5% higher than that obtained with TE using the M probe.

**Conclusions:**

Despite the measurement biases associated with the SWE systems, biases were not necessarily consistent, and they changed with the probes used and depth measured. The SWS of the viscoelastic phantom obtained using different modalities increased according to the shear wave frequency used.

## Introduction

Quantitative elastography has been widely used to evaluate liver fibrosis or differentiate malignant from benign lesions in various organs. According to Evidence-based Clinical Practice Guidelines for Liver Cirrhosis 2020 (3rd edition), sufficient evidence has already been demonstrated, and the usefulness of elastography, including ultrasound (US) and magnetic resonance elastography (MRE), is considered basic knowledge in the diagnosis of cirrhosis [1]. Although transient elastography (TE), which is a one-dimensional US-based elastography using a dedicated machine, has been the gold standard for the measurement of liver stiffness, shear wave elastography (SWE), including point SWE (pSWE) and two-dimensional color-coded SWE (2DSWE), which can be performed with a standard ultrasonography device, is more often performed clinically to evaluate the stiffness of various organs other than the liver. MRE has also been used to measure liver stiffness as a robust and reproducible method that can evaluate a large area [2]. There is a good correlation between shear wave speed (SWS) obtained with SWE and those obtained with TE and MRE, which were used as a reference [3–6].

Many studies have evaluated the diagnostic performance of US-based SWE (US-SWE) technology for the assessment of liver fibrosis or tumor malignancy and showed that US-SWE is a valid imaging biomarker for detection and, to some degree, staging of fibrosis [7] and differentiating between malignant and benign tumors [8, 9]. However, various optimal cutoff values have been proposed depending on the device, making it difficult to directly compare data obtained from different devices [10, 11]. Clinicians are uncertain whether they can use the cutoff value reported in studies using US systems other than their own. The major factor that causes bias is that the method of setting the region of interest (ROI) and the conditions for applying push pulses in SWS measurement vary among different SWE systems or among the software versions even in the same system.

Recently, some studies have evaluated the measurement bias between different US systems using phantoms and the human liver [12, 13]. Gilligan et al. [12] reported that the mean coefficients of variation (CV) across six 2DSWE systems were 2.2–4.4% in elastic phantoms and 6% in human liver, and Palmeri et al. [13] reported mean difference 95% confidence intervals (CIs) of 9.6% in elastic phantoms and 15.3% in viscoelastic phantoms. These studies evaluated variabilities using a convex probe, because SWS is most often used for the diagnosis of liver fibrosis. However, clinicians examining superficial organs, such as the breast and thyroid, want to know the variabilities in the case of use of linear probes.

The Quantitative Imaging Biomarker Alliance (QIBA), founded by the Radiological Society of North America, proposed a profile dedicated to standardizing MRE and SWS measurements by identifying bias in measurements and establishing a phantom suitable for characterization of data acquired from different systems [14, 15]. We have developed original multimodal phantoms for US and MRE and evaluated the agreement between SWS obtained from US and MRE [16]. We have also made a viscoelastic phantom of stiffness representing fibrotic liver or healthy thyroid that fulfills the QIBA acoustics specifications, which include speed of sound and attenuation coefficient [17, 18].

The aim of this study was to determine the bias in SWS measurements between commercially available US systems in a viscoelastic phantom and to compare them with the SWS obtained with TE and MRE.

## Materials and methods

### Phantom

A cylindrical viscoelastic phantom (diameter = 18 cm, height = 16 cm) for MR and US elastography was constructed using polyacrylamide (PAAm) gel [17]. It was composed of a three-dimensional network polymer and a large amount of liquid, which provided the MR signal. The storage modulus (*G*′) of the PAAm gel is mainly dependent on the acrylamide concentration, whereas the density of the three-dimensional network polymer is mainly dependent on the concentration of the cross-linker. Meanwhile, the loss modulus (*G*″) mainly depends on the ratio of glycerin to water. Aluminum oxide powder was added to the PAAm gel to generate US scattering. The tan *δ* (= *G*′/*G*″) of the liver was reported to be approximately 0.3 for both healthy volunteers and patients with liver fibrosis [19].

The phantoms for evaluation of the acoustic characteristics were cylinders with a diameter of 11 cm and height of 2 cm. The following substances and respective concentrations were used to fabricate the phantom: 13 wt% acrylamide, 1 wt% glycerin, 82 wt% water, 3 wt% aluminum oxide powder, and a total of less than 1 wt% of cross-linker, polymerization accelerator, and polymerization initiator. The procedure employed for fabricating the phantom was as follows. First, degassing was performed while mixing the acrylamide, glycerin, and cross-linker in water. Next, this mixture was cooled to 6 °C, the aluminum oxide powder and polymerization initiator were then mixed into it, and the polymerization accelerator was finally added [17, 18].

The acoustic characteristics, speed of sound, and attenuation coefficient were evaluated by analyzing the three-dimensional (3D) radio frequency (RF) signals acquired using a laboratory-made ultrasonic scanner with a single-element transducer with an aperture diameter of 6.35 mm and F-number of 5.25 (Model V310; Panametrics, WA, USA). The center frequency and − 6 dB bandwidth of the transducer were 5.0 MHz ± 2.0 MHz. The transducer was excited by a pulser/receiver (Model 5800; Panametrics, WA, USA) [20]. The energy level of the pulser was 12.5 µJ, and the cutoff frequencies of the low- and high-pass filters in the receiver were 1 and 35 MHz, respectively. The RF echo signals were digitized to 12 bits and sampled at 100 MHz using an oscilloscope (HDO6104; Lecroy, NY, USA). Two types of RF signals were acquired without a phantom (reference data) and with the phantom placed on an acrylic board in degassed water at 20 °C to calculate the speed of sound and the attenuation coefficient of the phantom. The transducer was mechanically scanned in the lateral plane with a scanning pitch of 100 µm using three-axis linear motor stages (MTN100CC; Newport, CA, USA). The collection volume for the 3D RF data was 4,096 samples in depth for each scan line, and 100 × 100 lines in the lateral plane. This gate length included the surfaces of the phantoms and the acrylic board. The speed of sound and attenuation coefficient for the phantom were evaluated using the reflector method [21]. The speed of sound $v$ was calculated from the time of flight based on the propagation peak-to-peak delay time as follows:

$$v = C_{ref} \left( {\frac{{t_{ref} - t_{p1} }}{{t_{p2} - t_{p1} }}} \right),$$

where *C*_*ref*_ is the speed of sound in degassed water at 20 °C; *t*_*ref*_ is the time of flight from the transducer to the reflector surface with no phantom; and *t*_*p1*_ and *t*_*p*2_ are the times of flight from the transducer to the phantom and reflector surfaces, respectively, with the phantom. The attenuation coefficient for the phantoms was calculated from the total attenuation *α*_total_ (*f*, *d*) [dB/cm] computed from the normalized power spectrum as follows:

$$\alpha_{{{\text{total}}}} \left( {f,d} \right) = \frac{8.686}{{2d}}\log_{{\text{e}}} \left( {\frac{{S_{p} \left( {f,d} \right)}}{{S_{ref} \left( {f,d} \right)}}} \right),$$

where ${S}_{p}\left(f,d\right)$ and ${S}_{ref}\left(f,d\right)$ are the power spectra of the echo signals from the phantom–reflector boundary with a phantom and the water–reflector boundary with no phantom at a frequency *f* and a depth *d*. The attenuation coefficient *α*_0_ was calculated as the slope of the linear equation ${\mathrm{\alpha }}_{total}\left(f,d\right)={a}_{0}f+b$ using the least-squares method with a − 6 dB bandwidth. The speed of sound and the attenuation coefficient were evaluated for each 3D scan line and averaged as 1560 ± 0.9 m/s and 0.5 ± 0.01 dB/cm/MHz, respectively. These acoustic characteristics satisfied the QIBA acoustics specification (speed of sound = 1540 ± 20 m/s, attenuation coefficient = 0.6 ± 0.2 dB/cm/MHz [14]). The viscoelasticity and acoustic properties of the phantom used in this study were adjustable, and the phantom could maintain physically stable properties for more than 18 months [17, 18].

### Ultrasound examination

The commercial ultrasound systems used were the following: Aplio i800 (Canon Medical Systems, Tokyo, Japan), ARIETTA 850 (FUJIFILM Healthcare, formerly Hitachi, Tokyo, Japan), LOGIQ E10 (GE Healthcare, Chicago, IL, USA), EPIQ Elite (Philips, Bothell, WA, USA), Aixplorer (SuperSonic Imagine, Aix-en-Provence, France), and ACUSON Sequoia (Siemens Healthineers, Mountain View, CA, USA). A FibroScan 430 mini (Echosens, Paris, France), a TE system, was used for comparison (Table 1).

**Table 1:** Probes, measurement modes, and methods of six ultrasound systems using acoustic radiation force and one system using transient elastography

Vendor	Equipment	Probe		Mode	SWE method
		Linear	Convex		
Canon medical systems	Aplio i800	i18LX5		Breast	2D
			i8CX1	Abdomen	2D
FUJIFILM healthcare (formerly Hitachi)	ARIETTA 850	L64		Thyroid	Point
			C252	Abdomen	Point
GE healthcare	LOGIQ E10	L2-9		Phantom	2D
		L2-9		Breast	2D
			C1-6	Phantom	2D
			C1-6	Abdomen	2D
Philips	EPIQ Elite	eL18-4		Breast	2D
			C5-1	Abdomen	2D
			C5-1	Abdomen	Point
SuperSonic imagine	Aixplorer	SL15-4		Breast	2D
		SL15-4		Phantom	2D
			XC6-1	Liver	2D
			XC6-1	Phantom	2D
Siemens healthineers	ACUSON Sequoia	10L4		Breast	2D
		10L4		Breast	Point
			5C1	Abdomen	2D
			5C1	Abdomen	Point
Echosens	FibroScan	M, XL			

*SWE* shear wave elastography, *2D* two-dimensional color-coded shear wave elastography, *point* point shear wave elastography

**Fig. 1:**
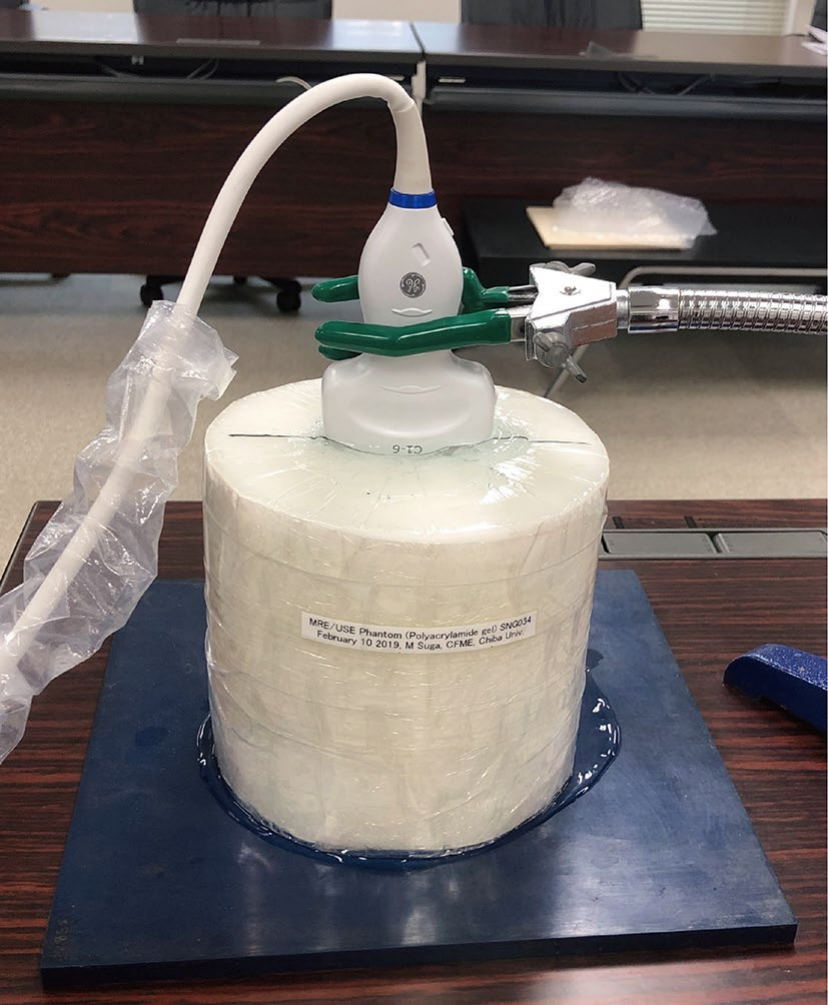
Phantom and probe settings. A probe was coupled to the phantom on an acoustic absorber using a jig

**Fig. 2:**
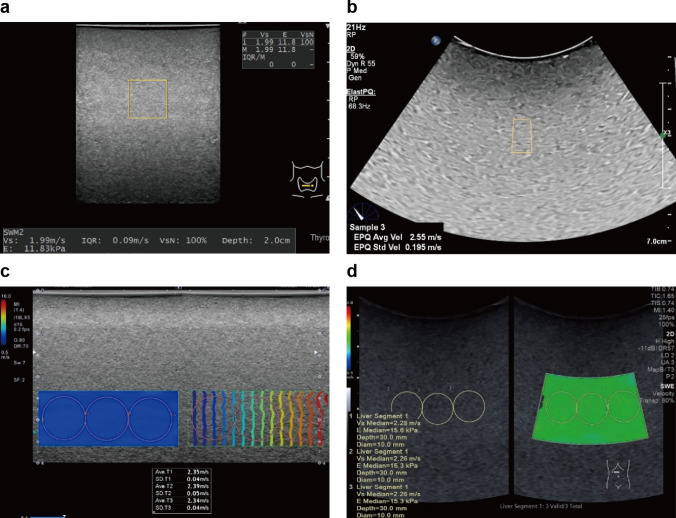
Example of setting of region of interest: point shear wave elastography using a linear probe (**a**) and a convex probe (**b**) and two-dimensional color-coded shear wave elastography using a linear probe (**c**) and a convex probe (**d**)

For each device, measurements were performed using linear and convex probes, with the probe coupled to the phantom on the acoustic absorber using a jig (Fig. 1). One ultrasound technician, with 26 years of experience in sonography and 14 years of experience assessing liver stiffness with US, fixed the probe to the phantom to give the same level of pressure as in clinical examinations. For devices that have both pSWE and 2DSWE applications or have a phantom mode in addition to the clinical mode, measurements were performed using all these modes. Hence, the SWS was measured using nine modes with linear probes and 10 modes with convex probes. SWS was measured at depths of 2 and 3 cm with a linear probe, as well as at depths of 3 and 4 cm with a convex probe (Table 1). SWS measurements were acquired at three ROIs, i.e., at the center of the phantom and at both sides of the phantom at each depth of the phantom (Fig. 2), and the measurement was repeated three times. Consequently, nine data points were obtained for one depth of each mode. A circular ROI with a diameter of 1 cm for 2DSWE and a rectangular ROI of fixed size for pSWE were used. For FibroScan, M and XL probes were used, and the measurements were repeated three times at the same three positions as other SWE systems. FibroScan displays liver stiffness as Young’s modulus, and they were converted to SWS using the following equation: Young’s modulus (Pa) = 3 × 10^3^ (kg/m^3^) × SWS^2^ (m^2^/s^2^), assuming a tissue density of 1 g/mL and a Poisson’s ratio of 0.5.

All ultrasound examinations were performed on a single day in April 2019 at the office of the Japan Society of Ultrasonics in Medicine at room temperature, which was maintained at 20 °C.

### Magnetic resonance elastography examination

MRE examination was performed using a 0.3-T open MRI system [FUJIFILM Healthcare (formerly Hitachi), Tokyo, Japan] and a custom-made cylindrical passive pneumatic driver connected to a commercial loudspeaker, based on a design used in a previous study [22]. The passive driver was positioned at the center of the top surface of the phantom. A spin-echo echo-planar MRE sequence [a motion-encoding gradient (MEG) was added to spin-echo echo-planar imaging using the sequence development environment of FUJIFILM Healthcare] was used to acquire coronal wave images. The imaging parameters were as follows: repetition time/echo time = 3000/67 ms, imaging matrix = 116 × 116, field of view = 348 × 348 mm^2^, slice thickness = 3.0 mm, and number of slices = 15. Three frequencies (continuous sinusoidal vibration) were selected for external excitation and MEG. The selected frequencies were 60 Hz (which is commonly used in clinical practice), 120 Hz (which is the highest frequency the system can measure), and 90 Hz (which is an intermediate frequency between them). *G*′ and *G*″ were calculated using a three-dimensional integral-type reconstruction formula [23]. MRE examination was performed 3 weeks after US examination, and the stiffness of the phantom was measured once.

**Fig. 3:**
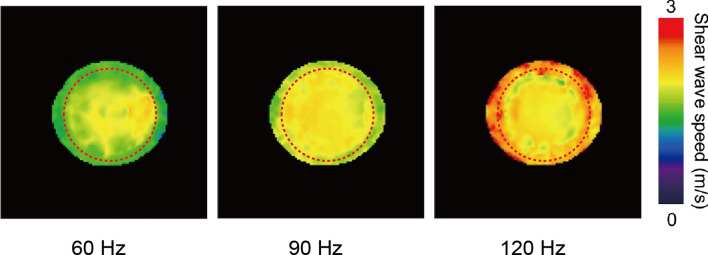
Shear wave speed images obtained with magnetic resonance elastography at excitation frequencies of 60, 90, and 120 Hz, and the region of interest (red dashed circle) for the mean and standard deviation calculations (color figure online)

Using the Voigt model for viscoelasticity, the SWS (m/s) for the MRE was calculated from *G*′ and *G*″ using the following equation [24]:

$${\text{SWS}} = \sqrt {\frac{{2\left( {G'^{2} + G''^{2} } \right)}}{{\rho \left( {G' + \sqrt {G'^{2} + G''^{2} } } \right)}}} ,$$

where *ρ* is the density of the material. An ROI was drawn in a circle with a diameter of 150 mm in the center of the phantom to avoid the peripheral area and thus avoid any error due to edge effects (Fig. 3).

### Statistical analysis

The median, interquartile range (IQR), and IQR/median of nine SWSs from each mode were assessed, and the grand mean, standard deviation (SD), and CVc (% = SD/grand mean × 100) of the median SWS of 9 or 10 modes were calculated. The percentage of difference in SWS between the different measurement depths or different modalities is defined as 2 × (SWS1 − SWS2)/(SWS1 + SWS2) × 100, where SWS1 and SWS2 are the SWSs of different depths or different modalities. Because MRE was measured only once for each MEG frequency, the SWS of the MRE was described, with the mean and SD indicating spatial variation. The total average SWS obtained with the US-SWE systems using all modes, probes, and depths was compared with the SWS obtained with MRE at several frequencies and TE using the M and XL probes. Statistical analysis was performed using SPSS version 25.0 (IBM Corp., Armonk, NY, USA).

## Results

**Fig. 4:**
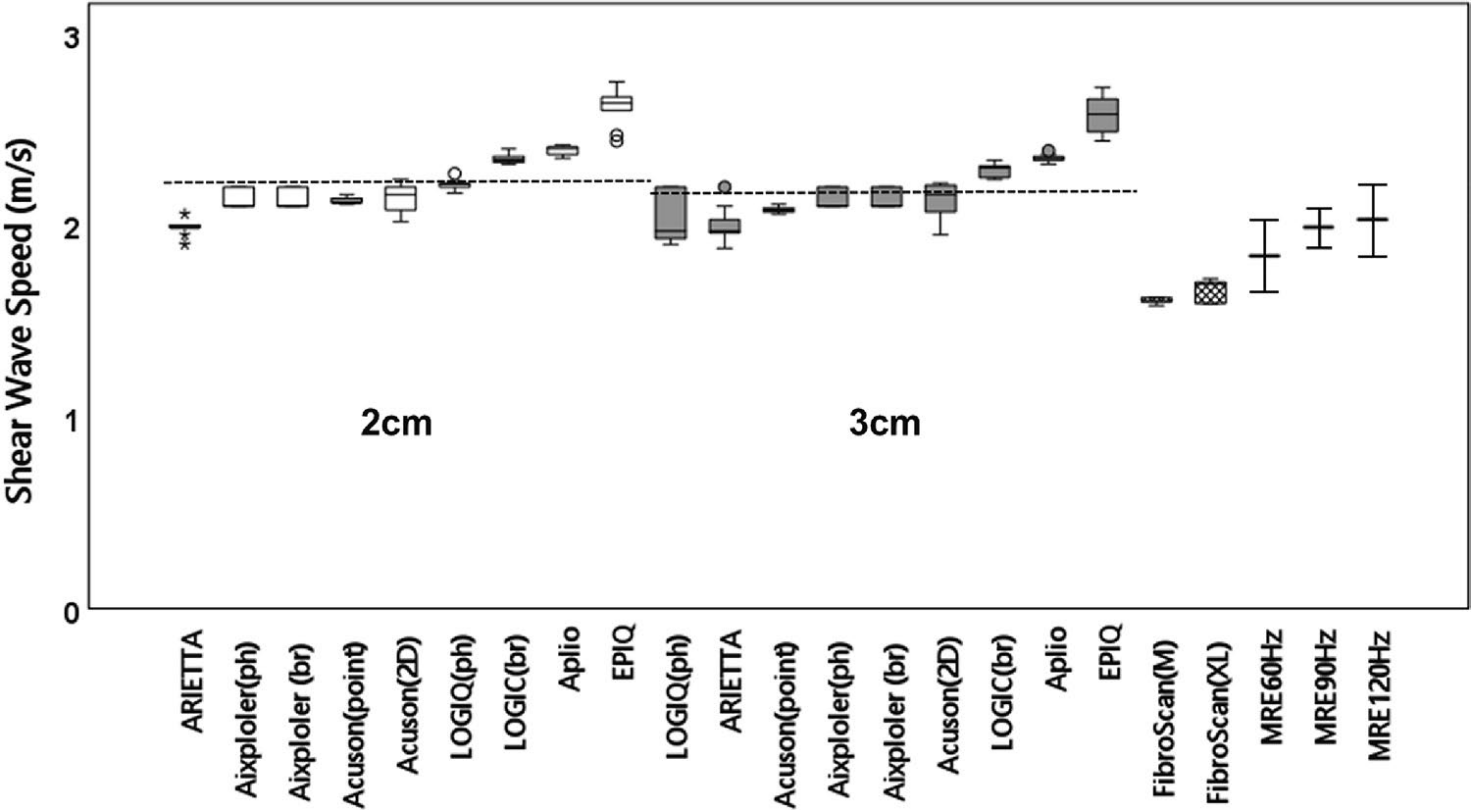
Box and whisker plots showing the SWS measurements using linear probes for nine measurement modes from six ultrasound systems at two focal depths (2 and 3 cm), transient elastography, and MRE. The horizontal dashed line represents the grand mean across all of the modes. The SWS of MRE was described, with the mean and standard deviation indicating spatial variation. *SWS* shear wave speed, *ph* phantom mode, *br* breast mode, *point* point shear wave elastography, *2D* two-dimensional color-coded shear wave elastography, *M* M probe, *XL* XL probe, *MRE* magnetic resonance elastography

**Fig. 5:**
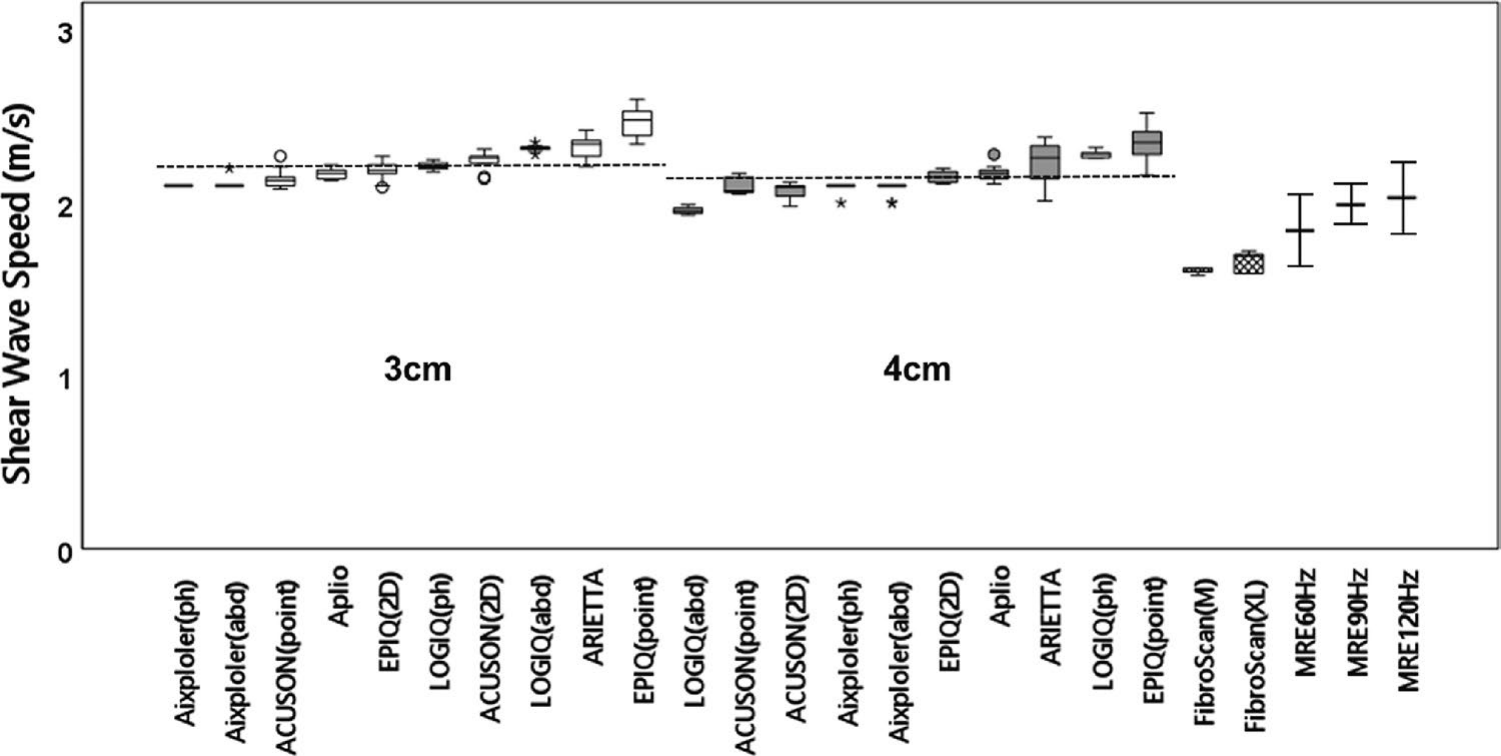
Box and whisker plots showing the SWS measurements using convex probes for 10 measurement modes from six ultrasound systems at two focal depths (3 and 4 cm), transient elastography, and MRE. The horizontal dashed line represents the grand mean across all of the modes. The SWS of MRE was described, with the mean and standard deviation indicating spatial variation. *SWS* shear wave speed, *ph* phantom mode, a*bd* abdominal mode, *point* point shear wave elastography, *2D* two-dimensional color-coded shear wave elastography, *M* M probe, *XL* XL probe, *MRE* magnetic resonance elastography

The *G*′, *G*″, and tan *δ* of the phantom on MRE measurements at 60 Hz were 3.1 kPa, 0.9 kPa, and 0.3, respectively. The median and IQR of the SWSs obtained from the SWE systems, TE, and MRE are summarized in Fig. 4 (linear probe) and Fig. 5 (convex probe). The horizontal dashed line in each plot represents the grand mean across all modes of the six SWE systems. The median SWS and IQR/median of each system and the grand mean, SD, and CV across all systems are summarized in Table 2. All IQR/median values were < 0.15, and the reproducibility of the measurement was considered to be high. The CVs were 9.0% and 9.2% at depths of 2 and 3 cm, respectively, for the linear probe and 5.4% and 5.6% at depths of 3 and 4 cm, respectively, for the convex probe. Most of the systems tended to yield a slower SWS in the deeper part than in the shallower part. The percentage of difference in SWS between the measurement depths in the same SWE systems was 2.3% ± 3.6% for the linear probe and 3.6% ± 5.7% for the convex probe. The total average SWS obtained with US-SWE systems using all modes, probes, and depths was 2.2 m/s. The median SWS and IQR/median of TE were 1.6 m/s and 0.013, respectively, with the M probe and 1.7 m/s and 0.066, respectively, with the XL probe. The SWSs obtained with MRE at 60, 90, and 120 Hz were 1.8 ± 0.2, 2.0 ± 0.1, and 2.1 ± 0.2 m/s, respectively. Among the three different modalities, the SWS obtained with US-SWE was the highest, followed by the SWS with MRE, which was higher for higher frequencies among the three MEG frequencies, and the SWS with TE was the lowest. The SWSs obtained with US-SWE were 19.9%, 9.4%, and 4.5% higher than the SWSs obtained with MRE at frequencies of 60, 90, and 120 Hz, respectively. They were also 31.5% and 26.1% higher than the SWSs obtained with TE using the M and XL probes, respectively.

**Table 2:** Shear wave speed using linear and convex probes of six ultrasound systems

Linear probes						
Equipment	Mode	SWE method	2 cm depth		3 cm depth	
			Median speed (m/s)	IQR/median	Median speed (m/s)	IQR/median
Aplio	Breast	2D	2.40	0.017	2.35	0.009
ARIETTA	Thyroid	Point	1.99	0.005	1.97	0.036
LOGIQ	Phantom	2D	2.21	0.009	1.97	0.137
	Breast	2D	2.34	0.004	2.30	0.026
EPIQ	Breast	2D	2.64	0.027	2.58	0.066
Aixplorer	Phantom	2D	2.10	0.048	2.10	0.048
	Breast	2D	2.10	0.048	2.10	0.048
ACUSON	Breast	Point	2.12	0.009	2.08	0.010
	Breast	2D	2.16	0.056	2.16	0.065
Grand mean of medians ± SD			2.23 ± 0.20		2.18 ± 0.20	
Coefficient of variation (%)			8.97		9.17	

Convex probe						
Equipment	Mode	SWE method	3 cm depth		4 cm depth	
			Median speed (m/s)	IQR/median	Median speed (m/s)	IQR/median
Aplio	Abdomen	2D	2.17	0.023	2.17	0.023
ARIETTA	Abdomen	Point	2.34	0.038	2.26	0.084
LOGIQ	Phantom	2D	2.21	0.014	2.28	0.013
	Abdomen	2D	2.32	0.004	1.95	0.015
EPIQ	Abdomen	Point	2.48	0.056	2.35	0.055
	Abdomen	2D	2.19	0.023	2.15	0.028
Aixplorer	Phantom	2D	2.10	0.000	2.10	0.000
	Liver	2D	2.10	0.000	2.10	0.000
ACUSON	Abdomen	Point	2.13	0.023	2.07	0.043
	Abdomen	2D	2.26	0.018	2.09	0.029
Grand mean ± SD			2.23 ± 0.12		2.15 ± 0.12	
Coefficient of variation (%)			5.38		5.58	

*SWE* shear wave elastography, *2D* two-dimensional color-coded shear wave elastography, *point* point shear wave elastography, *IQR* interquartile range, *SD* standard deviation

## Discussion

The present study, which quantified the bias of SWS measurements in a viscoelastic phantom across six different US systems and compared the SWS with those from TE and MRE, revealed that the SWS measurement bias was associated with US-SWE systems, and that the CV of their medians was up to 9.2% with a linear probe and 5.6% with a convex probe in viscoelastic phantom measurement. Because we measured SWS by fixing the probe to the phantom using a jig on an identical day in the same room, the factors of bias caused by aging of the phantom, room temperature, and operators were excluded.

A previous study of SWS measurement on an elastic phantom with 2DSWE using a convex probe reported mean CVs ranging from 2.2% to 4.4% and a CV of 3.4% in a phantom with stiffness comparable to that of our phantom [12]. Considering that we measured the viscoelastic phantom with pSWE and 2DSWE, our CV of 5.4–5.6% using a convex probe is considered to be close to the previous results. In another study with pSWE and 2DSWE using a convex probe, the CV was not specified, but it could be calculated from the mean difference 95% CI described in the figure of the Tukey mean difference plot: the CVs were calculated as 4.9% for elastic phantoms and 7.8% for viscoelastic phantoms [13]. Considering that the US systems used were different and the SWE software has been improved, the results with the viscoelastic phantom are comparable to our result using the convex probe.

Although the SWS bias associated with each system exhibited some tendencies, these biases were not necessarily consistent and changed with the probes used and depths measured. This is consistent with previous studies that reported that the bias changed across phantoms with different stiffnesses [12, 13]. Gilligan et al. [12] also disclosed vendor names in their paper, but the variability among the US systems was not completely consistent with those of our study. The bias due to measurement depth was a less significant confounding factor than system variability, which was also similar to a previous study [13].

The SWS is independent of the shear wave frequency content in elastic media, but it depends on the frequency in viscoelastic media [19]. Viscosity causes dispersion in the propagating shear waves, which means that the resultant SWS is dependent on the frequency content of the shear wave, with the higher frequency components of the shear wave propagating faster than the lower frequency components [13, 25]. The TE system generates a 50 Hz shear wave that is longitudinally polarized along the ultrasound axis, whereas 60, 90, and 120 Hz were used for the MRE excitation frequency in our study. The frequency of SWS used for pSWE/2DSWE has not been revealed by the vendors, but it is believed to be 100–500 Hz [26]. In our study, the SWS obtained with SWE was the highest, followed by MRE and TE, and in the order of MEG frequency among the MREs. Our result was in agreement with the expected theoretical result for viscoelastic phantom measurement and consistent with previous studies comparing SWE with TE or MRE in the measurement of a viscoelastic phantom and the liver [10, 17].

There are many other causes of measurement variability between US systems in addition to frequency. For example, the depth, shape, strength, and irradiation time of the push pulse; the three-dimensional spatial direction of shear wave propagation; the gate length of the detection pulse; the characteristics of filters; and the shape and size of the ROI cause variability. Nevertheless, manufacturers do not completely disclose the processing algorithms and scanner sequencing used in their devices, which is considered an underlying factor of the problem; therefore, standardization among systems is required [10, 13].

In our study, the bias of SWS among different SWE systems was larger with a linear probe than with a convex probe even at the same depth of 3 cm. This may be due to the difference in the number of SWE modes used with linear and convex probes in our study, but the difference in the processing techniques between linear and convex probes, as described above, might have affected the results. Few studies have evaluated measurement bias using a linear probe, and further studies are needed.

There are some limitations to our study. First, we measured only one viscoelastic phantom. As in previous studies, the measurement variability might be different for measurements of harder or softer phantoms. We have established a method to create phantoms with different viscoelasticities [18], and we will investigate the influence of various viscoelasticities in the future. In the case of a linear probe, evaluation of its use in a shallow area with a depth of about 1 cm, which is commonly used in clinical settings, is also being planned for future studies. Finally, further research is also needed to examine SWS bias in measurement of the liver and other organs across different systems.

## Conclusion

Viscoelastic phantom measurements made across different US-SWE systems had a CV of up to 9.2% with a linear probe and 5.6% with a convex probe. Although there were measurement biases associated with the different US-SWE systems, biases were not necessarily consistent, and they changed with the probes used and depth measured. The SWS of the viscoelastic phantom obtained using different modalities increased according to the shear wave frequency used. This study provides clinicians with insight into the range of bias in different devices and modalities.

## Declarations

### Research involved with human or animal participants

This article does not contain any studies with human participants performed by any of the authors.
